# Effect of a Ropy Exopolysaccharide-Producing *Bifidobacterium animalis* subsp. *lactis* Strain Orally Administered on DSS-Induced Colitis Mice Model

**DOI:** 10.3389/fmicb.2016.00868

**Published:** 2016-06-09

**Authors:** Claudio Hidalgo-Cantabrana, Francesca Algieri, Alba Rodriguez-Nogales, Teresa Vezza, Pablo Martínez-Camblor, Abelardo Margolles, Patricia Ruas-Madiedo, Julio Gálvez

**Affiliations:** ^1^Department of Microbiology and Biochemistry of Dairy Products, Instituto de Productos Lácteos de Asturias – Consejo Superior de Investigaciones CientíficasVillaviciosa, Spain; ^2^CIBER-EHD, Department of Pharmacology, ibs.GRANADA, Center for Biomedical Research, University of GranadaGranada, Spain; ^3^Geisel School of Medicine at DartmouthHanover, NH, USA; ^4^Universidad Autónoma de ChileSantiago, Chile

**Keywords:** *Bifidobacterium*, exopolysaccharide, mucoid, ropy, immune modulation, DSS-colitis, mouse model

## Abstract

Exopolysaccharide (EPS)-producing bifidobacteria, particularly *Bifidobacterium animalis* subsp. *lactis* strains, are used in the functional food industry as promising probiotics with purported beneficial effects. We used three isogenic strains of *B. animalis* subsp. *lactis*, with different EPS producing phenotypes (mucoid-ropy and non-ropy), in order to determine their capability to survive the murine gastrointestinal tract transit, as well as to evaluate their role in improving clinical outcomes in a chemically-induced colitis model. The three strains were able to survive in the intestinal tract of C57BL/6J mice during the course of the intervention study. Furthermore, the disease activity index (DAI) of the animal group treated with the ropy strain was significantly lower than of the DAI of the placebo group at the end of the treatment. However, no significant differences were found among the three strains. The analysis of several immune parameters, such as TNFα and IL-10 quantified in blood plasma and lymphocyte populations enumerated in mesenteric nodes, showed some significant variations among the four experimental animal groups. Remarkably, a higher capability of the ropy strain to increase regulatory T-cells in mesenteric lymphoid nodes was demonstrated, suggesting a higher ability of this strain to regulate inflammatory responses at mucosal level. Our data indicate that strains of *B. animalis* subsp. *lactis* producing EPS that confer a mucoid-ropy phenotype could represent promising candidates to perform further studies targeting intestinal inflammatory processes.

## Introduction

The human gut is one of the most densely populated ecosystems inhabited by a community of microorganisms that actively contributes to the health of the host. Microbiota establishes a symbiotic relationship with the host, but also their members must co-exist in a balanced state. Indeed, disturbances in microbiota composition (dysbiosis) and function, as well as in the microbiota-host cross-talk, are the basis of prevalent gastrointestinal diseases, and also of some extra-intestinal pathology (Tojo et al., 2014). Among others, microbiota shifts have been related to chronic inflammatory disorders, such as inflammatory bowel disease (IBD) (Huttenhower et al., 2014). Up to now, dietary interventions toward restoring the unbalanced microbiota, such as the oral administration of probiotics, have been a realistic approach for human applications (Reid et al., 2011; Collins, 2014). Hill et al. (2014) have recently reviewed the probiotic concept and they have adopted, with a minor grammatical correction, the definition “live microorganisms that, when administered in adequate amounts, confer a health benefit on the host,” previously proposed by the FAO and WHO organizations (FAO/WHO. Food and Agricultural Organization of the United Nations and World Health Organization, 2001). Research on probiotics has generated a vast amount of literature, mainly related to the characterisation of the probiotic potential of *Lactobacillus* and *Bifidobacterium*, the genera most commonly used for human consumption. However, only a small number of strains have been evaluated for the prevention or treatment of gastrointestinal disorders, such as IBD, diverse diarrheas, irritable bowel syndrome (IBS) or necrotising enterocolitis (NEC) (WGO. World Gastroenterology Organisation, 2011; Sanders et al., 2013). In relation to bifidobacteria, specific strains belonging to the species *Bifidobacterium breve, Bifidobacterium longum, Bifidobacterium bifidum*, and *Bifidobacterium animalis* subsp. *lactis*, have been tested in human intervention studies resulting in positive effects on gastrointestinal dysfunctions (Tojo et al., 2014).

*B. animalis* subsp. *lactis* is the species that has been most successfully introduced in food formulations, since it is able to deal with technological challenges during food manufacture (Prasanna et al., 2014). However, analysis of the available genomes shows a very low genetic variability among members of this subspecies (Milani et al., 2013). One of the features shared in all of their genomes is the presence of a cluster involved in the synthesis of exopolysaccharides (EPS) which are carbohydrate polymers surrounding the cell wall (Hidalgo-Cantabrana et al., 2014b). These EPS play a relevant role for the producing strain because they are involved in protection and niche colonization, but EPS also act as intermediaries in the dialog established between bacteria and host (Hidalgo-Cantabrana et al., 2014b). It has been proposed that EPS are able to interact with receptors located in the gut epithelium (Lebeer et al., 2010b) and they could act as effector molecules eliciting different immune responses (Hidalgo-Cantabrana et al., 2012).

In recent years our group has been working on the study of biological properties of EPS synthesized by bifidobacteria. One of the *B. animalis* subsp. *lactis* strains from our collection displayed a mucoid-ropy phenotype, denoted by the formation of a long filament from the colony, and it was able to synthesize a high molecular weight (HMW)-EPS. Recently, working with three recombinant strains, we demonstrated the involvement of a mutation (C> 266 >T) in *wzz* gene in the occurrence of this phenotype and in the synthesis of the HMW-EPS fraction. This gene encodes a hypothetical membrane anchored protein, with a predicted large soluble domain, which is theoretically involved in the chain length determination of the polymer (Hidalgo-Cantabrana et al., 2015). The acquisition of the ropy phenotype favored the *in vitro* survival and adhesion of the recombinant strain to the gut environment. In turn, this strain also elicited an *in vitro* and *ex vivo* anti-inflammatory cytokine profile (Hidalgo-Cantabrana et al., 2015). Thus, in a step forward the current work aims to *in vivo* evaluate the probiotic potential of the ropy *B. animalis* subsp. *lactis* Balat_1410^S89L^ checking its capability to counteract an inflamed state of colitis chemically induced by dextran sodium sulfate (DSS) administration.

## Materials and methods

### Bifidobacterial strains and preparation of lyophilized bacteria in milk

Three isogenic EPS-producing *B. animalis* subsp. *lactis* strains from the IPLA-CSIC collection were used in this study to test their probiotic capabilities in an *in vivo* model. Based on the type strain DSM10140, recombinant strains were obtained from a previous study (Hidalgo-Cantabrana et al., 2015): strain ΔBalat_1410 (DSM10140 lacking the gene Balat_1410 complemented with pAM1, non ropy), strain Balat_1410 (strain ΔBalat_1410 complemented with pAM1 + Balat_1410, non ropy), and Balat_1410^S89L^ (strain ΔBalat_1410 complemented with pAM1 + Balat_1410^S89L^, ropy). Glycerol stocks kept at −80°C were plated on agar MRSC [MRS Difco (BD, Biosciences, San Diego, CA) containing 0.25% L-cysteine-HCl (Sigma-Chemical Co., St. Louis, MO)] supplemented with erythromycin (2.5 μg/ml) (MRSCE). Plates were incubated at 37°C under anaerobic conditions (80% N_2_, 10% CO_2_, 10% H_2_) in an MG500 chamber (Don Whitley Scientific, West Yorkshire, UK). A single colony per strain was used to inoculate 50 ml MRSCE broth and after 20 h this culture was used to inoculate 2% (v/v) 1 L fresh medium. After 20 h incubation, cultures were centrifuged, washed once with PBS and resuspended in 100 ml tindalized 11% skimmed milk (Difco). Then, bacterial suspensions in milk were lyophilized for 48 h in the Virtis Freezemobile 12EL equipment (SP Scientific, NY). Each lyophilized bifidobacterial stock was tested for bacterial viability and enumeration by counting in agar-MRSCE and incubated under standard conditions. For oral administration, each lyophilized bifidobacteria was resuspended daily in water (at 5 × 10^9^ cfu/ml) and 100 μl was administered per mouse.

### Animals, experimental designs and sample collection

All studies were carried out following the Directive 2010/63/EU of the European Parliament and the Council on the protection of animals used for scientific purposes (Directive 2010/63/EU, 2010). The experimental protocol was approved by the Ethical Committee of Laboratory Animals of the University of Granada (Spain) (Permit Number CEEA-2010-286). Male C57BL/6J mice (7–9 weeks old, approximately 20 g) were obtained from Janvier Labs (St Berthevin Cedex, France) and kept under conventional conditions with a standard pelleted diet and sterilized water for 1 week before beginning the experiments.

#### Bifidobacteria survival study

Animals (32 mice) were randomly assigned to four experimental groups with 8 mice per group: three bifidobacterial groups, each orally receiving 100 μl/day (5 × 10^8^ cfu) of the corresponding milk-bifidobacterial suspension in water, and one placebo (control) group which received 100 μl/day of skimmed milk. Placebo and bifidobacteria suspensions were orally administered for 9 days by means of an intra-gastric cannula. Every day, the drinking water and food intake was measured for each group of mice, whereas the body weight was measured individually for each animal. Stool samples were taken, in duplicate, at group level every 2 days, including the first and last day. At the end of the intervention period (day 10), each mouse was anesthetized with isoflurane and then, the animals were sacrificed by cervical dislocation by expert and qualified persons, according to the Federation of European Laboratory Animal Science Associations (FELASA). The colon was excised, its content was collected and its length and weight was measured after washing with PBS. Stool and colon-content samples were immediately processed as described in the next section.

#### Dextran sodium sulfate (DSS) induced colitis study

**Figure 1:**
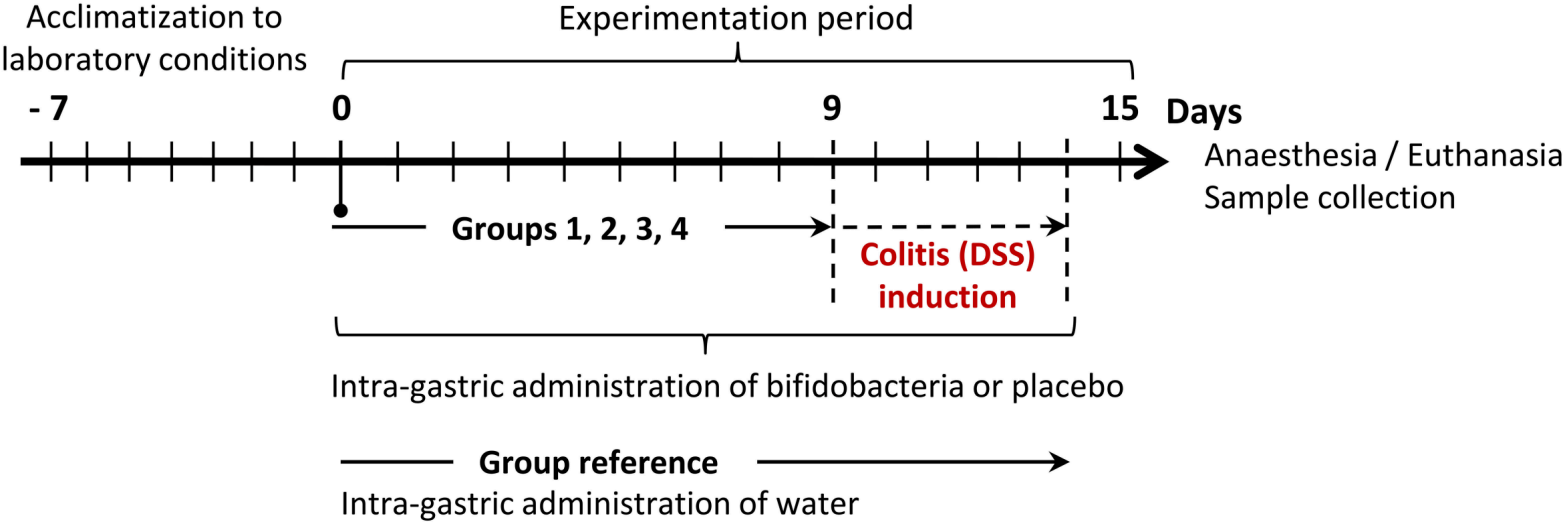
**Experimental design to check the capability of EPS-producing *B. animalis* subsp. *lactis* strains to counteract the dextran sodium sulfate (DSS)-induced colitis**. Group 1 (placebo) daily receiving 100 μl skimmed milk and Groups 2–4 daily receiving 100 μl milk with 5 × 10^8^ cfu bifidobacteria: group 2, strain ΔBalat_1410 (DSM10140 lacking the gene Balat_1410 complemented with pAM1, non ropy); group 3, strain Balat_1410 (strain ΔBalat_1410 complemented with pAM1 + Balat_1410, producing a non-ropy EPS); group 4, strain Balat_1410^S89L^ (strain ΔBalat_1410 complemented with pAM1 + Balat_1410^S89L^, producing a ropy EPS). Reference group (non DSS-induced colitis, non-placebo/bifidobacteria fed group) daily receiving 100 μl water.

Mice (40 animals) were randomly assigned to five groups, each with 8 animals, as described in Figure 1. The reference (non-colitic, non-placebo/bifidobacteria fed) group received daily, through an intra-gastric cannula, 100 μl water during the 15-day experimental period. The other four groups were fed with bifidobacteria, or a placebo, and treated with DSS. During the 15 days of the experimental period these four groups received a placebo (100 μl/day milk, group 1), or different bifidobacterial strains (100 μl/day 5 × 10^8^ cfu milk-bifidobacterial suspensions in water, for groups 2, 3, and 4). After 9 days of bifidobacteria/placebo feeding, colitis was induced in the four groups by adding DSS (3%, w/v, 36–50 kDa, MP Biomedicals, CA) in the drinking water (Mähler et al., 1998). This DSS-treatment was kept for a period of 6 days unless the application of humane end-point, as defined below, was needed. At the end of the experimental period (day 15) animals were anesthetized and a blood sample was directly extracted from the heart using heparinized tubes. The blood plasma was obtained by centrifugation (9,300*xg*, 4°C, 20 min). The animals were then euthanatized in order to collect colon and colon-content, as described above, as well as mesenteric lymphoid nodes. Samples were immediately processed or stored at −20°C until their analysis.

Drinking water and food intake was measured at group level each day, whereas body weight, stool consistency and the presence of gross blood in feces were evaluated daily and scored (Table 1) for each mouse during the experimental period. The disease activity index (DAI) was calculated as previously reported by Cooper et al. (1993): total score [body weight decrease + stool consistency + rectal bleeding] / 3. The ulcerative colitis (UC) disease is considered for DAI ≥ 1.5 and the humane endpoint was fixed at DAI = 3. Stool samples were taken at group level every 2 days to check viability of bifidobacteria.

**Table 1:** **Disease activity index (DAI) score used to evaluate the DSS-induced colitis. DAI index was calculated as total score (body weight decrease + stool consistency + rectal bleeding) divided by 3**.

**Score**	**Body weight decrease (%)**	**Stool consistency**	**Rectal bleeding**
0	< 1	Normal	Normal
1	1–5		
2	5–10	Loose stools	
3	10–20		
4	>20	Diarrhea	Gross bleeding

*The humane end-point was established at DAI = 3*.

### Microbial quantification in fecal samples

#### Bifidobacterial quantification by counting

Stool samples, collected in both studies as previously indicated, were homogenized in PBS (0.1 g/ml) using a Heidolph (Heidolph Instruments GmbH, Schwabach, Germany) stirrer for 1 min. Then, 100 μl was used to obtain serial dilutions in PBS which were plated onto the surface of TOS propionate agar medium (Merck, Darmstadt, Germany), supplemented with lithium-mupirocin as recommended by the manufacturer, to selectively enumerate total bifidobacteria. Additionally, agar lithium-mupirocin-TOS was supplemented with 2.5 μg/ml erythromycin (TOS+Ery) which is the selective marker of the plasmid present in the three *B. animalis* subsp. *lactis* strains under study. All plates were incubated at 37°C in an anaerobic jar using Oxoid-Anaerogen™ (Thermo Fisher Scientific Inc., Waltham, MA) sachets to generate anaerobic conditions for 72 h.

#### Bifidobacterial identification by 16S rDNA amplification

Several colonies were picked up from TOS+Ery plates of each of the four experimental groups belonging to the bifidobacteria survival study. The DNA from a total of 67 colonies was isolated using the GenElute Bacterial Genomic DNA kit (Sigma) according to the manufacturer's instructions, but adding a prior step of bacterial lysis with mutanolysin and lysozyme. Primer uses were: pA/pH (Edwards et al., 1989). The PCR mixture (final volume 50 μl) was: 1 μl chromosomal DNA, 0.20 μM of each primer, 200 μM dNTPs (Amersham Bioscience, Upsala, Sweden) and 2.5 U Taq DNA-polymerase (Eppendorf, Hamburg, Germany). After an initial cycle at 95°C, the PCR amplification consisted of 30 cycles: 95°C for 1 min, 55°C for 50 s and 68°C for 2 min, and ended with a final extension step of 68°C for 10 min. Reactions were carried out in the thermal cycler UnoCycler (VWR International Eurolab S.L., Barcelona, Spain). Purification and sequencing of the amplicons were performed in Macrogen (Seoul, Korea). The free Chromas 1.45 software (Technelysium Pty Ltd., Australia) was used to process sequences which were compared to those held in the GenBank database (http://www.ncbi.nlm.nih.gov/genbank) using the BLASTn tool.

### Gene expression analyses in colonic samples by reverse transcriptase qPCR

After colon excision, PBS-washed colonic tissue was sectioned in small fragments which were placed in RNAlater stabilization reagent (Qiagen GmbH, Hilden, Germany) and then stored at −80°C until RNA extraction. Total RNA was isolated using RNeasy Mini Kit (Qiagen) according to the manufacturer's recommendations. Purity and RNA concentration were determined with the NanoDropTM 2000 spectrophotometer. Then, 3 μg RNA was reverse transcribed to obtain cDNA using oligo(dT) primers and reagents from Promega (Promega, Southampton, UK) and the TProfessional basic thermocycler (Biometra GmbH, Goettingen Germany).

The amplification was performed in an Eco™ Real-Time PCR System (Illumina, San Diego, CA USA) using 20 ng of cDNA and specific primers (Table S1). The 2^−ΔΔCt^ method was used to normalize expression results (Livak and Schmittgen, 2001). The values of the house-keeping glyceraldehyde-3-phosphate dehydrogenase (GAPDH) gene were first used to normalize the values obtained for each of the genes under study. These values were additionally normalized to those obtained in the reference group, thus this group had a normalized value of 0. Finally the relative expression of each gene was calculated as 2^−ΔΔCt^, thus the reference group had the relative expression value of 1.

### Cellular population in mesenteric lymphoid nodes

The mesenteric lymphoid nodes were carefully mashed with wet slides to decrease friction and the solutions were filtered through a 70 μM cell strainer. Cells were isolated, counted and plated on anti-CD3 (clone17A2, eBioscience, San Diego, CA, USA) and anti-CD28 (clone 37.51, eBioscience)—coated plates for FcγR blocking. Then, the cells were transferred to polystyrene tubes for surface staining with anti-CD4 (PerCP-Cy™5.5, clone RM4-5 BD Pharmigen™, Franklin Lake, NU, USA), anti-CD45 (APC-eFluor®780, clone 30-F11, eBioscience) and Viability Dye (eFluor®660, eBioscience) for 15 min at 4°C in the dark. The cells were then fixed, permeabilized with the Fixation/Permeabilization kit (eBioscience) and intracellular staining was done with anti-Foxp3 (PE, clone FJK-16s, eBioscience) for 30 min at 4°C in the dark. Data collection was performed using a flow cytometer CANTO II (BD Biosciences San Diego, CA, USA).

### Cytokine levels of blood samples

The levels of tumor necrosis factor (TNF)-α and interleukin (IL)-10 cytokines in blood plasma were quantified using the mouse TNFα Quantikine and mouse IL-10 Quantikine ELISA kits (R&D Systems, Abingdon, UK) following the manufacturer's instructions.

### Statistical analyses

All considered variables were distributed normally (Kolmogorov-Smirnov test was used to check normality); thus, they are described as mean ± standard deviation. Robust Student-Welch or ANOVA tests were used in order to check the equality among groups for all variables, and *post-hoc* T3 de Dunnet or LSD tests were used to compare each two groups. Standard linear regression models were employed to compute the size of the effect (95% confidence interval) of the different treatments on the DAI; to do this, crude data were adjusted by the initial conditions (DAI score the first day of DSS-induced colitis) and the placebo group was used as reference. Differences below 0.05 were considered statistically significant. All analyses were made with the free statistical software R (www.r-project.org).

## Results and discussion

### *B. animalis* subsp. *lactis* strains are able to colonize the murine gut

Orally administered probiotics should survive the upper gastrointestinal tract (GIT) transit and reach the colon alive, where they should persist for a certain time in order to be able to exert their beneficial effect. Therefore, an animal experimentation procedure was done to assess the viability of the three *B. animalis* subsp. *lactis* strains under study aiming to check whether the presence of the ropy HMW-EPS surrounding Balat_1410^S89L^ strain could exert a protective role. Additionally, although *B. animalis* has QPS (qualified presumption of safety) status (EFSA BIOHAZ Panel, 2013), we evaluated the lack of toxicity of our strains, at this oral-dose administered to the animals, by measuring animal behavior and wellbeing parameters. In this regard, no differences (*p* > 0.05) were found in water and food intake, body weight, stool consistency or colon anatomy between the placebo (skimmed milk-fed) and each of the bifidobacteria-fed groups (Table 2). These results support the fact that the three bifidobacterial strains used in this study had no side effects at the concentration level administered to this murine model.

**Table 2:** **Mean and standard deviation of general parameters measured in four experimental groups of mice to evaluate the absence of toxicity of the bifidobacterial strains administered for 10 days at dose of 5 × 10^8^ cfu per mouse and day**.

**Experimentation group**	**Food intake^a^**	**Drink water^a^**	**Body weigh increase**^b^		**Ratio weight/length of colon^c^**
	**(g/mouse)**	**(ml/mouse)**	**(g)**	**(%)**	
Placebo (skim milk)	2.22 ± 0.39	2.52 ± 0.48	0.73 ± 0.17	3.24 ± 0.75	0.024 ± 0.005
Strain ΔBalat_1410	2.49 ± 0.23	2.79 ± 0.34	0.73 ± 0.16	3.20 ± 0.70	0.024 ± 0.009
Strain Balat_1410	2.24 ± 0.29	2.68 ± 0.45	0.85 ± 0.25	3.84 ± 1.13	0.025 ± 0.008
Strain Balat_1410^S89L^	2.18 ± 0.31	2.84 ± 0.51	0.66 ± 0.18	2.89 ± 0.78	0.026 ± 0.006

*No significant statistical differences (p > 0.05) were found among the four experimental groups after one-way ANOVA study*.

a *Values obtained for the whole mice group for each day of experimental procedure*.

b *body weight at day 10 subtracting the body weight measured at day 0 (27.71 ± 1.11) before the experimental procedure*.

c *colon weight and length determined after 10 days of experimental procedure*.

**Figure 2:**
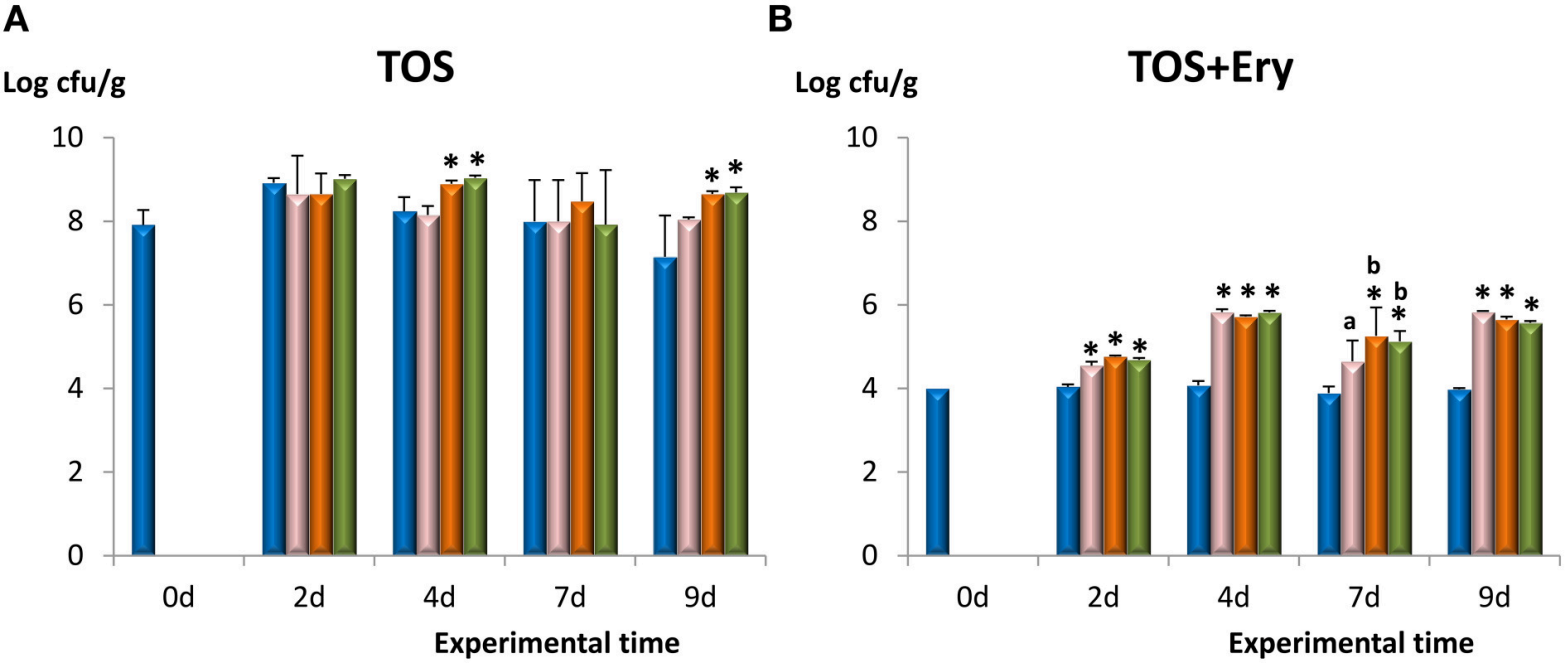
**Counts in agar-TOS to enumerate total bifidobacterial population (A) and agar-TOS+Ery (supplemented with 2.5 μg/ml erythromycin, the antibiotic marker of strains under study) to enumerate erythromycin-resistant bacteria (B) in fecal homogenates obtained from four experimental groups of mice along the experimental period**. Placebo group (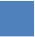), feed *B. animalis* subsp. *lactis* ΔBalat_1410 (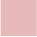), feed *B. animalis* subsp. *lactis* Balat_1410 (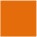), and feed *B. animalis* subsp. *lactis* Balat_1410^S89L^ (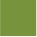). Within each treatment day: statistical differences with respect to the control group are marked with an asterisk (*p* < 0.05) and differences among the three bifidobacterial groups are denoted with different letters (*p* < 0.05).

The bifidobacterial population in the fecal samples was enumerated using agar-TOS (Figure 2A) which is a selective medium for bifidobacteria growing in milk products. After 2 days of administration, the bifidobacterial levels increased in all groups about 1 log unit, including the placebo receiving skimmed milk. However, during prolonged treatment the population of bifidobacteria in the placebo group tended to decline to reach the initial values, whereas counts in the three bifidobacteria-fed groups remained stable. Similarly, counts obtained in TOS+Ery, which is the medium containing the selective marker erythromycin of the plasmid present in the three strains under study, were significantly higher (*p* < 0.05) in the three bifidobacterial-fed groups with respect to the placebo (Figure 2B). Levels of erythromycin-resistant bacteria in the microbiota of placebo-fed mice were around 4 log units and remained unaltered for the duration of the experimentation time. After 2 days of administration, levels of erythromycin-resistant bifidobacteria increased (on average) 0.7 log cfu/g in the mice receiving bifidobacteria; the highest count increase (1.7 log cfu/g) was reached after 4 days, remaining at similar values until the end of the experimentation period. In general, no statistical differences among the three bifidobacteria-fed groups were found and, additionally, the colonies chosen to partially sequence the gene 16S rDNA were identified as members of *Bifidobacterium* genus; those isolated from Balat_1410^S89L^ fed animals displayed a ropy phenotype as well (see Table S2). Altogether, these results show that our bifidobacterial strains were able to survive as members of the intestinal microbiota of mice for at least 9 days, following a daily intake of 5 × 10^8^ cfu. The increase in total bifidobacteria observed in this study could be related to the ability of EPS-producing *B. animalis* subsp. *lactis* to raise the number of other bifidobacterial species in the intestinal microbiota of rodents as previously described (Salazar et al., 2011). This effect could be related to the ability of members from this microbial community to use EPS as fermentable substrates (Salazar et al., 2009). However, the increase of erythromycin resistant members in feces is indicative of a higher survival rate in the gut of the specific strains used in this work. The *in vivo* performance of the three strains is rather similar, since there was no statistical difference in the number of bifidobacteria present in the fecal samples among the three groups of mice. Thus, it seems that the presence of HMW-EPS on the surface of the ropy Balat_1410^S89L^ strain does not confer an additional protection of survival in mice gut. Fanning et al. (2012) found that fecal counts (made in Reinforced *Clostridium* agar with 50 mg/L mupirocin) of mice fed with EPS^+^ *B. breve* UCC2003 were significantly higher than those obtained with two non-EPS producing mutant strains. Thus, these authors conclude that the presence of an EPS on the surface of the wild type *B. breve* was involved in its higher survival rate in the mice gut. On the contrary, *in vivo* studies carried out with different EPS-producing *Lactobacillus johnsonii* strains showed that mutants with deletion of genes from the *eps* cluster had an increased residence time in the animal's gut (Denou et al., 2008; Horn et al., 2013). It has also been shown that the loss of EPS production in *Lactobacillus rhamnosus* GG improves the *in vitro* adhesion of the strain to Caco2 cells (Lebeer et al., 2009); however, the presence of the EPS surrounding the wild type GG strain improves its *in vivo* performance allowing the strain to persist for longer periods because the EPS protects against the antimicrobial peptide LL-37 (Lebeer et al., 2010a). The intrinsic characteristics of EPS, such as composition and size, seem to promote different effects on bacterial survival under gut challenges. In some cases, polymers could prevent the exposure of molecules from the bacterial envelope involved in adhesion, but EPS can also act as a shield against host immune defense (Hidalgo-Cantabrana et al., 2012).

### The ropy *B. animalis* subsp. *lactis* balat_1410^S89L^ reduces DSS-induced damage

As mentioned above, *in vitro* and *ex vivo* tests (using human tissues) showed different immune modulation capabilities of the three isogenic bifidobacteria, the ropy strain Balat_1410^S89L^ having an anti-inflammatory profile (Hidalgo-Cantabrana et al., 2015). To expand on these observations, we wanted to address whether this predicted anti-inflammatory ability is effective *in vivo*, modifying the immune response in a model of gut inflammation such as mice with DSS-induced colitis. This is a well-established animal model of acute mucosal inflammation that has been used for several decades (Wirtz et al., 2007) and it has been validated as a model for the translation of mice data to humans (Melgar et al., 2008).

**Figure 3:**
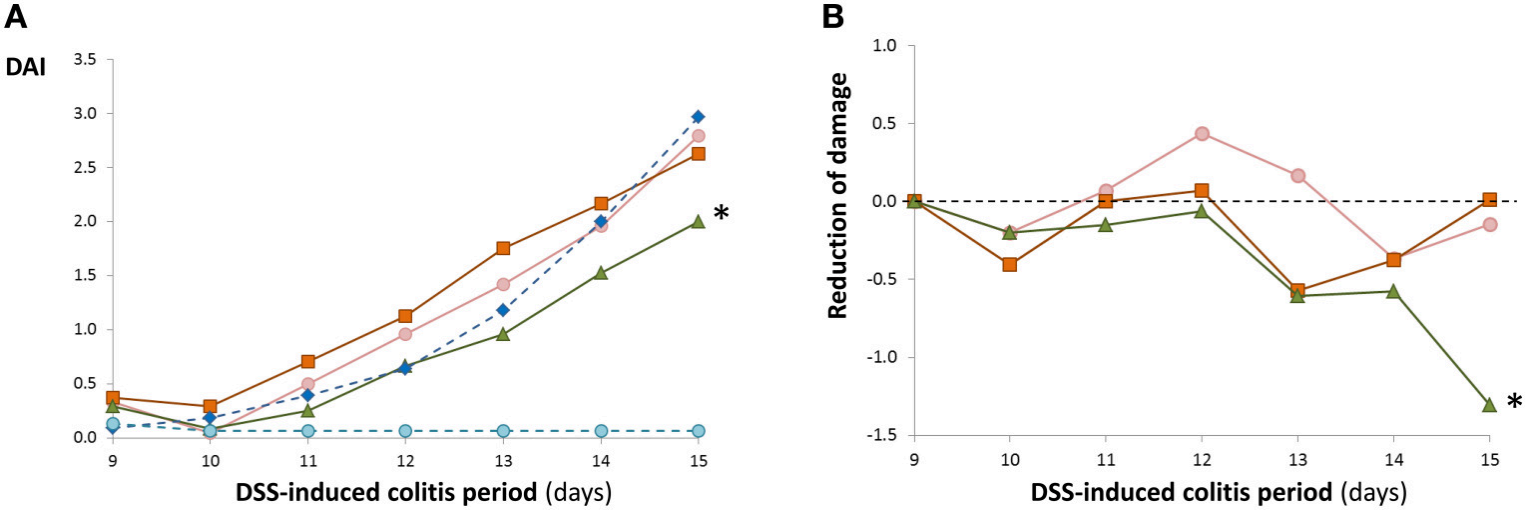
**Mean values of the disease activity index (DAI, Table 1) calculated for each experimental group**. The coefficient of variation (SD/mean) percentage of these values varied between 9 and 70%. Each bifidobacteria-fed group was compared with the placebo throughout the DSS-treatment and statistical differences (*p* < 0.05) are marked with an asterisk **(A)**. The DAI score of each bifidobacterial group was adjusted, as indicated in material and methods, to values of placebo at the first day of DSS-treatment (value = 0). Negative values indicate a higher reduction in the disease severity with respect to the placebo. These adjusted data were compared with placebo during the 5 days of DSS-treatment and statistical differences (*p* < 0.05) are marked with an asterisk **(B)**. Reference group (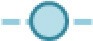); DSS-induced colitis groups: placebo (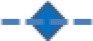), feed *B. animalis* subsp. *lactis* ΔBalat_1410 (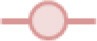), feed *B. animalis* subsp. *lactis* Balat_1410 (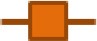), feed *B. animalis* subsp. *lactis* Balat_1410^S89L^ (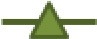).

In our experimental procedure, four groups of mice were pre-treated with an oral administration of bifidobacteria, or placebo, for 9 days before the administration of DSS in drinking water, which was continued for an additional 6 days (Figure 1). As was expected, in accordance with results shown in the previous section, the pre-feeding for 9 days in the four groups did not modify the wellbeing and behavior parameters of the mice. This is also consistent with the absence of significant variations in the disease activity index (DAI) score of these groups (values lower than 0.5, data not shown). The DAI score was kept close to 0 in the non-colitic, non-placebo/ bifidobacteria-fed (reference) group during all experimental procedures. In the four colitic groups, after the second day (day 11) of DSS treatment, the DAI values increased continuously (Figure 3A). The maximum score was obtained at the end of the experimental procedure, after 6 days of DSS colitic induction. In general, no statistical differences were detected on each day of treatment among the placebo and the three bifidobacteria-fed groups during DSS-treatment; the exception was the group fed with the strain Balat_1410^S89L^, which showed a significantly (*p* < 0.05) lower DAI score than the placebo group at day 15. These results were confirmed when DAI data were adjusted by means of linear regression models taking into consideration the DAI of placebo group each day of DSS-induction (Figure 3B). This model allowed us to determine the effect of the treatments on the DAI with respect to the placebo (value = 0). Thus, negative values of the adjusted DAI indicate a reduction in the severity of the disease in comparison with the placebo group. The highest reduction in adjusted DAI obtained for *B. animalis* subsp. *lactis* Balat_1410^S89L^ fed animals at the end of the treatment was mainly associated with a reduction in diarrhea and rectal bleeding. This led to a 44% reduction in the severity of the disease with respect to the placebo (*p* < 0.007), whereas, in the other two bifidobacteria-fed animals this reduction was not statistically significant, although the reduction of the illness was 23.2% for the strain ΔBalat_1410 and 16.7 % for the strain Balat_1410. In spite of the remarkable differences among the three strain-fed groups (disease severity in Balat_1410^S89L^ was reduced by almost two and three-fold compared to ΔBalat_1410 and Balat_1410, respectively), these differences were not deemed statistically significant. Therefore, we cannot ascertain that the protective effect against DSS-induced colitis exerted by strain *B. animalis* subsp. *lactis* Balat_1410^S89L^ is attributed to the HMW-EPS surrounding the bacteria. Some authors have reported the capability of probiotic strains to reduce the DAI index of animals under DSS-induced colitis. However, a mixture of several bacteria, or combinations of bacteria-bioactive compounds, are often tested, thus the positive effect cannot be assigned to a single bacterium (Dai et al., 2013). Only a few reports have shown the efficacy of single bifidobacterial strains (Hayes et al., 2014; Zheng et al., 2016).

### Different *B. animalis* subsp. *lactis* strains elicit specific host responses

**Figure 4:**
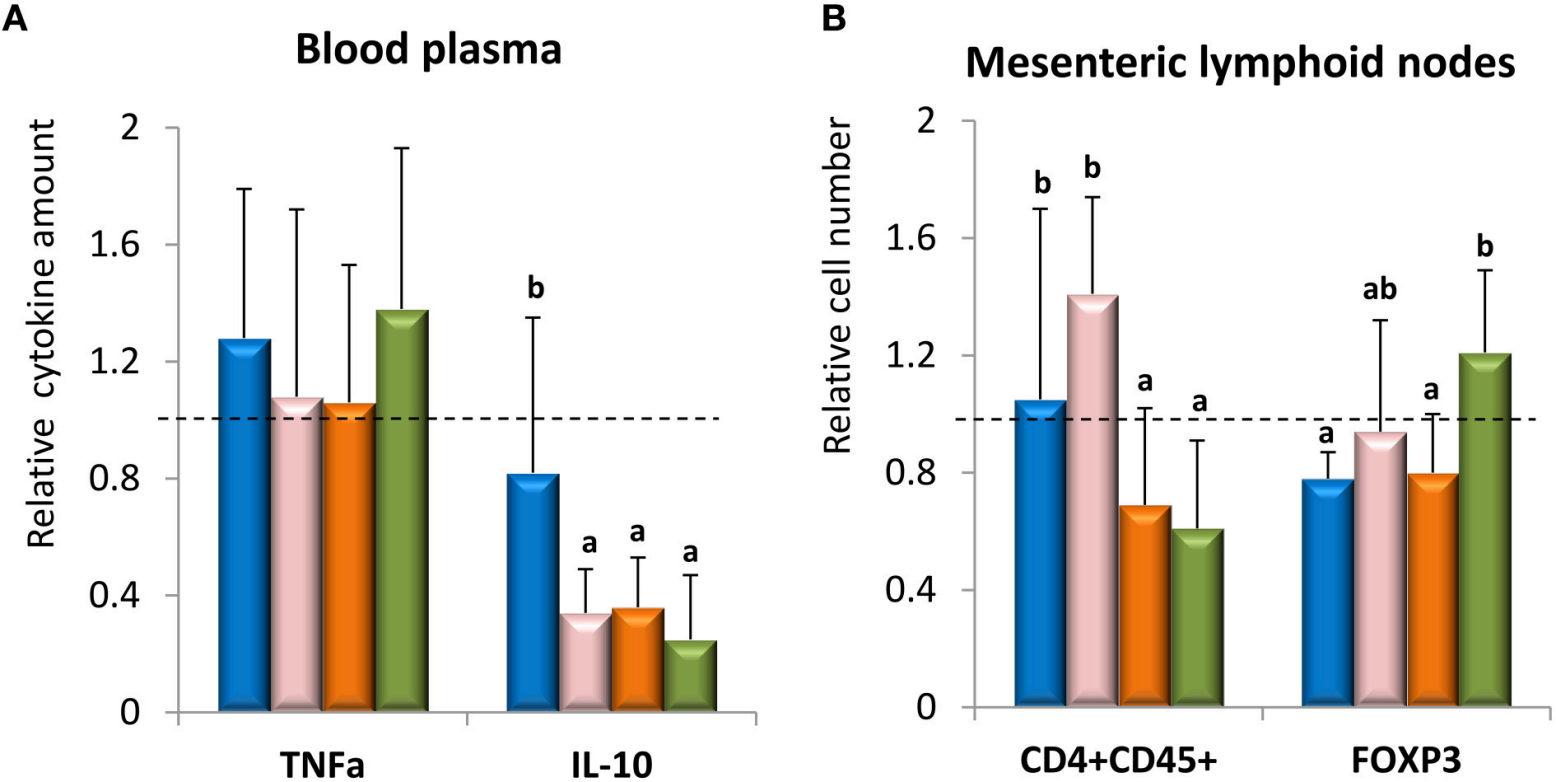
**Mean values of relative TNFα and IL-10 cytokines measured in blood samples (A) and relative number of CD4^+^CD25^+^ (T helper cells) and FOXP_3_ (T regulatory cells) quantified in mesenteric lymphoid nodes (B)**. Samples were collected after 6-days of DSS-treatment. Values of each DSS-colitic animal group were referred to as the average of those obtained in the reference group (value = 1). Placebo group (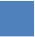), feed *B. animalis* subsp. *lactis* ΔBalat_1410 (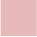), feed *B. animalis* subsp. *lactis* Balat_1410 (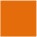), feed *B. animalis* subsp. *lactis* Balat_1410^S89L^ (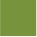). Within each parameter: statistical differences among the four groups are denoted with different letters (*p* < 0.05).

In order to understand the mechanism(s) involved in the attenuation of the DSS-colitis by the strain Balat_1410^S89L^ several immune parameters, targeting adaptive as well as innate responses, were measured in different tissues. In our study, the expression of several genes related to the reinforcement of the intestinal epithelium barrier, such as those coding for chemokines, proteins of tight junctions and mucins, showed no statistical variations (*p* > 0.05) among the four groups of animals (Table 3). Only the non-ropy EPS-producing strain Balat_1410 was able to slightly induce (*p* < 0.05) the expression of Mcp1, a chemokine able to attract monocytes, as well as the secretory protein TFF3, which is synthetized by goblet cells and acts to stabilize the mucus layer. Thus, it seems that, based on these results of gene expression, our strains were not able to modify the epithelial barrier in a remarkable way. However, it was reported that probiotics are able to reinforce the intestinal barrier function by multiple mechanisms (Lebeer et al., 2010a; Rao and Samak, 2013). The efficacy of *Bifidobacterium infantis* BB-02 to reduce intestinal injury in NEC was due to its ability to keep claudin-4 and occludin located in the tight-junction of bifidobacteria-fed mice (Bergmann et al., 2013). It was also demonstrated that bioactive factors released by *B. infantis* (one of the strains present in the VSL#3 mixture) were also able to reduce gut permeability by acting upon tight junction proteins (Ewaschuk et al., 2008). Similarly *B. bifidum* OLB6378 also favored the location of tight-junction and adherent-junction proteins, also being able to induce the synthesis of mucin-3, TFF3, and IL-6 (Khailova et al., 2009). These works, among others, show that bifidobacteria are able to improve the gut barrier function in a strain dependent manner. Regarding expression of cytokine genes in colonic tissue, statistical differences (*p* < 0.05) were detected only for pro-inflammatory IL-1β and IFNγ genes and the anti-inflammatory IL-10 (Table 3); in general, the two first genes were expressed at a lower rate in the placebo group with respect to the three bifidobacteria-fed groups, whereas IL-10 gene had significantly lower expression rate only for the ropy strain Balat_1410^S89L^. Thus, it seems that this strain induced the expression of genes that could lead to a mild pro-inflammatory response, since the ratio TNFα/IL-10 was significantly higher than that obtained for the other three groups (data not shown). A similar pro-Th1 profile was obtained with wild-type EPS-producing *B. animalis* subsp. *lactis* strains (one of them also producing a ropy HMW-EPS) co-cultivated *in vitro* with PBMC and GALT (gut associated lymphoid tissue) cells isolated from naïve rats (Hidalgo-Cantabrana et al., 2014a). Indeed, in the current study the concentration of these cytokines measured in blood plasma (Figure 4A) gives TNFα/ IL-10 ratio values of 1.69 ± 0.97 for placebo, 4.32 ± 2.15 for strain ΔBalat_1410, 2.77 ± 1.85 for strain Balat_1410, and 10.28 ± 9.15 for strain Balat_1410^S89L^. The ratio obtained for the animals fed with the ropy strain was significantly (*p* < 0.05) higher than that obtained for the other three groups of mice. However, it is worth noting that none of the three strains, or the placebo, was able to induce a strong immune systemic response since the values of both cytokines were near (or lower) than value 1, which is the value of the reference animal group. Thus, this suggests that our strains, or the skimmed milk (placebo) used as a protectant for bifidobacteria delivery, did not contribute to increase the inflammatory process induced by DSS.

**Table 3:** **Relative expression of genes related to immunity of intestinal mucosa in colonic tissue**.

**Parameter^a^**		**Animal experimentation groups**				***p*-value**
		**Placebo**	**Strain ΔBalat_1410**	**Strain Balat_1410**	**Strain Balat_1410^S89L^**	
Cytokines	TNFα	8.93 ± 5.42	8.64 ± 3.14	10.48 ± 3.99	7.57 ± 1.89	0.752
	IL-10	3.85 ± 1.68^b^	3.85 ± 1.63^b^	2.19 ± 0.72^a^,^b^	1.75 ± 0.26^a^	**0.041**
	IL-17	0.93 ± 0.29	0.86 ± 0.29	0.76 ± 0.23	0.83 ± 0.26	0.662
	IL-1β	10.98 ± 7.12^a^	11.88 ± 2.89^a^	28.94 ± 7.36^b^	22.98 ± 3.94^b^	**0.001**
	IL-6	10.61 ± 8.42	11.88 ± 7.14	12.98 ± 6.62	15.27 ± 2.80	0.800
	IL-12	0.91 ± 0.41	1.39 ± 1.00	1.52 ± 0.63	0.93 ± 0.28	0.168
	TGFβ	2.30 ± 0.98	3.19 ± 2.30	1.96 ± 1.12	1.03 ± 0.75	0.309
	IFNγ	0.77 ± 0.69^a^	2.07 ± 1.72^b^	2.44 ± 0.94^b^	2.57 ± 0.95^b^	**0.029**
Chemokines	iCAM-1	1.91 ± 0.35	1.81 ± 0.93	1.48 ± 0.81	1.52 ± 0.38	0.552
	Mcp1	5.12 ± 1.28^a^	4.93 ± 2.74^a^	11.41 ± 5.08^b^	4.39 ± 1.87^a^	**0.001**
Tight junctions	Occludin	0.66 ± 0.22	0.45 ± 0.22	0.62 ± 0.22	0.46 ± 0.09	0.106
	ZO-1	0.69 ± 0.26	0.50 ± 0.24	0.60 ± 0.27	0.48 ± 0.21	0.314
Secretory protein	TFF3	1.54 ± 0.56^a^	2.41 ± 1.15^a^	3.67 ± 1.29^b^	1.76 ± 0.37^a^	**0.002**
Mucins	Muc1	2.92 ± 1.40	3.99 ± 2.00	6.11 ± 3.23	4.52 ± 2.42	0.148
	Muc2	0.63 ± 0.38	0.42 ± 0.21	0.49 ± 0.29	0.52 ± 0.12	0.436
	Muc3	0.88 ± 0.32	1.24 ± 0.66	1.69 ± 1.00	1.64 ± 1.09	0.158
Enzymes	Mmp9	2.52 ± 0.74	3.31 ± 1.60	2.13 ± 0.90	2.78 ± 1.25	0.342
	INOS	3.37 ± 2.46	6.52 ± 3.94	10.08 ± 8.63	5.06 ± 1.97	0.096

*Values in each DSS-colitic group were referred to the average of those obtained in the control (no-colitic, value = 1) one. P-values from one-way ANOVA tests carried out among four groups; those values within the same row that do not share a common letter are statistically different according to the mean comparison LSD (p < 0.05)*.

a *IL-, interleukin-; TNFα, tumor necrosis factor α; TGF-β, transforming growth factor β; IFNγ, interferon γ; MUC-, mucin-; TFF-3, trefoil factor 3; ZO-1, zonula occludens 1; MMP-9, matrix metallopeptidase 9; iNOS, inducible nitric oxide synthase; ICAM-1, intercellular adhesion molecule*.

b *The statistical differences among the four bifidobacterial groups are denoted with different letters (p < 0.05)*.

*Bold values underlines statistical differences according to one-way ANOVA test*.

Immune cells isolated from the lymphoid nodes associated with the gastrointestinal tract were quantified by flow cytrometry and significant (*p* < 0.05) variations among the four experimental groups were detected (Figure 4B). Mice orally receiving the ropy Balat_1410^S89L^ or the non-ropy Balat_1410 strains presented a significant reduction in the population of CD4^+^/CD45^+^ cells, which are the markers for helper T-lymphocytes. The number of this population was even slightly lower than that of the reference group (value 1). In addition, the group receiving the strain Balat_1410^S89L^ was the only one significantly different from the placebo regarding the number of cells expressing Foxp3^+^, which acts as a regulatory factor for differentiation and function of regulatory T-lymphocytes. Besides, values of Foxp3^+^ cells in this group of animals, which also showed a reduced severity in DAI score, were slightly higher than the value of the animal reference group. Thus we suggest that the oral administration of the ropy strain could favor the recruitment of Treg cells to the intestinal mucosa, which could lead to a decrease in the severity of the damage caused by DSS. Jeon et al. (2012) showed *in vivo* that *B. breve* Yakult strain was able to ameliorate T cell-dependent colitis in mice through the induction of Treg cells toward producing IL-10. Similar results were obtained with a mixture of five probiotic strains and a mice model mimicking human Crohn's disease (Kwon et al., 2010). These authors also conclude that the efficacy of probiotics was due to an enrichment of CD4^+^ Foxp3^+^ Treg in the inflamed area. In recent years our group has been working with two models of isogenic, ropy and non-ropy EPS-producing *B. animalis* subsp. *lactis* strains: wild-types and the recombinant strains used in the current work. The results regarding the immune response that both types of strains could elicit are consistent when immune cells from the same origin are used. That is, all types of strains are able to induce a mild pro-inflammatory (Th1) response when a murine (rats or mice) model is used, either by *in vitro* (Hidalgo-Cantabrana et al., 2014a) or *in vivo* (the current work) approaches. However, the same EPS-producing strains tested *in vitro* or *ex vivo*, with immune cells and colonic tissue from human origin, had a reduced capability to elicit an immune response, or displayed an anti-inflammatory cytokine profile (López et al., 2012; Hidalgo-Cantabrana et al., 2015). These results underline the strong influence of the biological model used to test immune capability of strains with probiotic potential. Variations obtained with both models could be related to the differences in composition and function of the microbiota inhabiting the human and mouse intestines (Wos-Oxley et al., 2012).

Finally, due to the absence of statistical differences among the three strains we cannot conclude without doubt that the protective effect of DAI score promoted by strain Balat_1410^S89L^ was due to the synthesis of the HWM-EPS. However, we can hypothesize that the ropy HMW-EPS (produced in higher amounts by this strain) could act as a physical barrier, like a biofilm, protecting from chemical insults and, consequently, reducing the severity of the mucosa damage. In this regard, we have previously shown that the EPS purified from the wild ropy strain *B. animalis* subsp. *lactis* A1dOxR was able to *in vitro* counteract the cytotoxic effect of bacterial toxins upon humans (Caco2 monolayers) or animals (rabbit erythrocyte) cells (Ruas-Madiedo et al., 2010). Synthesis of EPS by bifidobacteria in intestinal environmental conditions has not been proved to date; however, production of EPS by commensal *Bacteroides fragilis* in the gut of mice has recently been proved (Geva-Zatorsky et al., 2015). Thus, this fact would support our hypothesis that the HMW-EPS from strain *B. animalis* subsp. *lactis* Balat_1410^S89L^ forms an EPS-biofilm protecting, to a certain degree, the intestinal epithelium from DSS-induced damage.

## Conclusion

In this work we have shown that the ropy strain *B. animalis* subsp. *lactis* Balat_1410^S89L^ was able to protect mice from injury caused by DSS. To date, we have not found modifications in the expression of genes related to the reinforcement of the intestinal barrier that could explain the attenuation of chemically-induced damage; the most relevant immune parameter that could be related to this fact is the high capability of strain Balat_1410^S89L^ to induce Treg cells in mesenteric lymphoid nodes which, in turn, could lead to a localized reduction of the inflammation at mucosal level. In spite of the only difference among the three isogenic recombinant strains tested in this study being the production of a ropy HMW-EPS, we cannot without doubt attribute the capability to reduce the DSS-induced damage to the polymer produced by Balat_1410^S89L^.

There is a lack of consistency in the immune modulation capability of the ropy HMW-EPS-producing *B. animalis* subsp. *lactis* strains due to the high influence of the biological model used. However, the ability of the strain Balat_1410^S89L^ to survive in the mice gut and the absence of adverse effects on the animals, together with previous *in vitro* and *ex vivo* evidence upon human cells, makes it feasible that the ropy (wild-type) *B. animalis* subsp. *lactis* could be a good candidate to check its anti-inflammatory ability in patients suffering from intestinal inflammation.

## Author contributions

JG, AM, and PR contributed with the conception, experimental design and results interpretation of this study. CH carried out all experiments, some of them performed with the collaboration of FA, AR, and TV. PM was in charge of the statistical analyses. PR was in charge of writing the drafted manuscript. All authors performed a critical revision of the manuscript and approved the final version.

### Conflict of interest statement

The authors declare that the research was conducted in the absence of any commercial or financial relationships that could be construed as a potential conflict of interest.
