# Supplementary material for: Effect of a Ropy Exopolysaccharide-Producing Bifidobacterium animalis subsp. lactis Strain Orally Administered on DSS-Induced Colitis Mice Model
Source: Front Microbiol. 2016 Jun 9;7:868. doi: 10.3389/fmicb.2016.00868 (PMC4900019; doi:10.3389/fmicb.2016.00868)
Supplement: Supplementary file 1 [file Table1.DOCX]

**Table S1** Primers used in this work to analyse the gene expression in mouse colonic tissue by reverse transcriptase quantitative PCR.

| **Target ^a^** | **Sequence (5’→ 3’)** | **Annealing** | **Reference** |
| --- | --- | --- | --- |
| GAPDH | **F**: CCATCACCATCTTCCAGGAG | 60ºC | Garrido-Mesa et al., 2011 |
| (reference) | **R**: CCTGCTTCACCACCTTCTTG |  |  |
| IL-1β | **F**: TGATGAGAATGACCTCTTCT | 55 ºC | Garrido-Mesa et al., 2011 |
|  | **R**: CTTCTTCAAAGATGAAGGAAA |  |  |
| IL-17 | **F**: CCTGGGTGAGCCGACAGAAGC | 60 ºC | Algieri et al., 2014 |
|  | **R**: CCACTCCTGGAACCTAAGCAC |  |  |
| IL-12 | **F**: CTGGTGCAAAGAAACATGGA | 60 ºC | Garrido-Mesa et al., 2015 |
|  | **R**: TGGTTTGATGATGTTCCTGA |  |  |
| IL-6 | **F**: TAGTCCTTCCTACCCCAATTTCC | 60 ºC | Garrido-Mesa et al., 2015 |
|  | **R**: TTGGTCCTTAGCCACTCCTTC |  |  |
| IL-10 | **F**: TCCTTAATGCAGGACTTTAAGGG | 56 ºC | Garrido-Mesa et al., 2015 |
|  | **R**: GGTCTTGGAGCTTATTAAAAT |  |  |
| TNFα | **F**: AACTAGTGGTGCCAGCCGAT | 56 ºC | Garrido-Mesa et al., 2011 |
|  | **R**: CTTCACAGAGCAATGACTCC |  |  |
| TGF-β | **F**: GCTAATGGTGGACCGCAACAAC | 60 ºC | Zhu et al., 2012 |
|  | **R**: CACTGCTTCCCGAATGTCTGAC |  |  |
| IFNγ | **F**: GAACTGGCAAAAGGATGGTGA | 60 ºC | Algieri et al., 2014 |
|  | **R**: TGTGGGTTGTTGACCTCAAAC |  |  |
| MUC-1 | **F**: GCAGTCCTCAGTGGCACCTC | 60 ºC | Garrido-Mesa et al., 2015 |
|  | **R**: CACCGTGGGGCTACTGGAGAG |  |  |
| MUC-2 | **F**: GATAGGTGGCAGACAGGAGA | 60 ºC | Utrilla et al., 2015 |
|  | **R**: GCTGACGAGTGGTTGGTGAATG |  |  |
| MUC-3 | **F**: CGTGGTCAACTGCGAGAATGG | 60 ºC | Garrido-Mesa et al., 2011 |
|  | **R**: CGGCTCTATCTCTACGCTCTC |  |  |
| TFF-3 | **F**: CCTGGTTGCTGGGTCCTCTG | 60 ºC | Wlodarska et al., 2011 |
|  | **R**: GCCACGGTTGTTACACTGCTC |  |  |
| ZO-1 | **F**: GGGGCCTACACTGATCAAGA | 56 ºC | Garrido-Mesa et al., 2011 |
|  | **R**: TGGAGATGAGGCTTCTGCTT |  |  |
| Occludin | **F**: ACGGACCCTGACCACTATGA | 56 ºC | Garrido-Mesa et al., 2015 |
|  | **R**: TCAGCAGCAGCCATGTACTC |  |  |
| MMP-9 | **F**: TGGGGGGCAACTCGGC | 60 ºC | Garrido-Mesa et al., 2011 |
|  | **R**: GGAATGATCTAAGCCCAG |  |  |
| iNOS | **F**: GTTGAAGACTGAGACTCTGG | 56 ºC | Garrido-Mesa et al., 2011 |
|  | **R**: GACTAGGCTACTCCGTGGA |  |  |
| ICAM-1 | **F**: GAGGAGGTGAATGTATAAGTTATG | 60 ºC | Garrido-Mesa et al., 2011 |
|  | **R**: GGATGTGGAGGAGCAGAG |  |  |
| MCP-1 | **F**: CAGCTGGGGACAGAATGGGG | 63 ºC | Garrido-Mesa et al., 2011 |
|  | **R**: GAGCTCTCTGGTACTCTTTTG |  |  |

**^a^** GAPDH, glyceraldehyde-3- phosphate dehydrogensase; IL-, interleukin-; TNFα, tumour necrosis factor α; TGF-β, transforming growth factor β; IFNγ, interferon γ; MUC-, mucin-; TFF-3, trefoil factor 3; ZO-1, zonula occludens 1; MMP-9, matrix metallopeptidase 9; iNOS, inducible nitric oxide synthase; ICAM-1, intercellular adhesion molecule 1.

**References**

Algieri F, Rodriguez-Nogales A, Garrido-Mesa N, Zorrilla P, Burkard N, Pischel I, Sievers H, Benedek B, Feistel B, Walbroel B, Rodriguez-Cabezas MA, Galvez J. 2014. Intestinal anti-inflammatory activity of *the Serpylli herba* extract in experimental models of rodent colitis. J Crohn's and Colitis **8**:775–788.

Garrido-Mesa N, Utrilla P, Comalada M, Zorrilla P, Garrido-Mesa J, Zarzuelo A, Rodríguez-Cabezas MA, Gálvez J. 2011. The association of minocycline and the probiotic *Escherichia coli* Nissle 1917 results in an additive beneficial effect in a DSS model of reactivated colitis in mice. Biochem Pharmacol **82**:1891–1900.

Garrido-Mesa J, Algieri F, Rodriguez-Nogales A, Utrilla MP, Rodriguez-Cabezas MA, Zarzuelo A, Ocete MA, Garrido-Mesa N, Galvez J. 2015. A new therapeutic association to manage relapsing experimental colitis: doxycycline plus *Saccharomyces boulardii*. Pharmacol Res **97**:48–63.

Utrilla MP, Peinado MJ, Ruiz R, Rodriguez-Nogales A, Algieri F, Rodriguez-Cabezas MA, Clemente A, Galvez J, Rubio LA. 2015. Pea (*Pisum sativum* L.) seed albumin extracts show anti-inflammatory effect in the DSS model of mouse colitis. Mol Nutr Food Res **59**:807-819.

Wlodarska M, Willing B, Keeney M, Menendez A, Bergstrom KS, Gill N, Russell SL, Vallance BA, Finlay BB. 2011. Antibiotic treatment alters the colonic mucus layer and predisposes the host to exacerbated *Citrobacter rodentium*-induced colitis. Infect Immun 79:1536-1545.

Zhu Ch, Wang Q, Zhou J, Liu H, Hua F, Yang H, Hu Z. 2012. The mineralocorticoid receptor-p38MAPK-NFκB or ERK-Sp1 signal pathways mediate aldosterone-stimulated inflammatory and profibrotic responses in rat vascular smooth muscle cells. Acta Pharmacol Sinica **33**: 873–878.
