# Supplementary material for: Effect of a Ropy Exopolysaccharide-Producing Bifidobacterium animalis subsp. lactis Strain Orally Administered on DSS-Induced Colitis Mice Model
Source: Front Microbiol. 2016 Jun 9;7:868. doi: 10.3389/fmicb.2016.00868 (PMC4900019; doi:10.3389/fmicb.2016.00868)
Supplement: Supplementary file 2 [file Table2.DOCX]

**Table S2** Identification by partial sequencing of 16S rDNA of 67 colonies picked from the surface agar TOS+Ery plates used to analyse fecal samples corresponding with the placebo and the three bifidobacteria-feed groups.

| **Experimental mouse group** | **Number of colonies** | **Identification** (number, % identity) | **Ropy phenotype** |
| --- | --- | --- | --- |
| Placebo (skim milk) | 21 | *Lb. acidophilus* (1, 99 %) *Lb. reuteri* (16, **≥** 95 %) *Lb. rhamnosus* (4, 95 %) | No No No |
| *B. animalis* subsp. *lactis* Strain ∆Balat_1410 Strain Balat_1410  Strain Balat_1410^S89L^ | 17 13 16 | *B. animalis* subsp. *lactis* (17, 95 %) *B. animalis* subsp. *lactis* (13, 95 %) *B. animalis* subsp. *lactis* (16, 95 %) | No No Yes (16) |
